# Household Food Insecurity as a Predictor of Stunted Children and Overweight/Obese Mothers (SCOWT) in Urban Indonesia

**DOI:** 10.3390/nu10050535

**Published:** 2018-04-26

**Authors:** Trias Mahmudiono, Triska Susila Nindya, Dini Ririn Andrias, Hario Megatsari, Richard R. Rosenkranz

**Affiliations:** 1 Department of Nutrition, Faculty of Public Health, Universitas Airlangga, Surabaya 60115, East Java, Indonesia; triska.nindya@fkm.unair.ac.id (T.S.N.); dien_ra@yahoo.com (D.R.A.); 2 Department of Health Promotion and Education, Faculty of Public Health, Universitas Airlangga, Surabaya 60115, East Java, Indonesia; hario.megatsari@gmail.com; 3 Department of Food, Nutrition, Dietetics and Health, Kansas State University, Manhattan, KS 66506, USA; ricardo@ksu.edu

**Keywords:** food security, HFIAS, double burden of malnutrition, child stunting, Indonesia

## Abstract

**(1) Background**: The double burden of malnutrition has been increasing in countries experiencing the nutrition transition. This study aimed to determine the relationship between household food insecurity and the double burden of malnutrition, defined as within-household stunted child and an overweight/obese mother (SCOWT). **(2) Methods**: A cross-sectional survey was conducted in the urban city of Surabaya, Indonesia in April and May 2015. **(3) Results**: The prevalence of child stunting in urban Surabaya was 36.4%, maternal overweight/obesity was 70.2%, and SCOWT was 24.7%. Although many households were food secure (42%), there were high proportions of mild (22.9%), moderate (15.3%) and severe (19.7%) food insecurity. In a multivariate logistic regression, the household food insecurity access scale (HFIAS) category significantly correlated with child stunting and SCOWT. Compared to food secure households, mildly food insecure households had the greatest odds of SCOWT (adjusted odds ratio (aOR) = 2.789; 95% confidence interval (CI) = 1.540–5.083), followed by moderately food insecure (aOR = 2.530; 95% CI = 1.286–4.980) and severely food insecure households (aOR = 2.045; 95% CI = 1.087–3.848). **(4) Conclusions**: These results support the hypothesis that the double burden of malnutrition is related to food insecurity, and the HFIAS category is a predictor of SCOWT.

## 1. Introduction

One of the primary public health problems of the 21st century is the obesity epidemic, affecting over half a billion people worldwide [1]. In 2008, an estimated 1.46 billion adults were overweight (body-mass index (BMI) ≥ 25 kg/m^2^), with 205 million men and 297 million women among them categorized as obese (BMI ≥ 30 kg/m^2^) [2]. Obesity does not solely affect developed countries; the developing countries of the world have also experienced great increases in prevalence [1]. Data from the World Health Organization (WHO) show that developing countries in Africa and Southeast Asia will soon face the levels of overweight currently prevalent in developed countries such as the USA [3]. In 2013, the prevalence of overweight and obesity among Indonesian males was 20%, while the prevalence among females was already hitting 35% [4].

At the opposite end of the nutritional spectrum, the prevalence of undernutrition remains a major public health problem. Although the United Nations’ efforts to combat malnutrition through the Millennium Development Goals (MDGs) have been progressing toward attainment [5], almost half of all deaths among children under the age of five years are still attributable to undernutrition [6]. The World Health Organization (WHO) recently reported that approximately 45% of all deaths among children under the age of five years were associated with under-nutrition in 2017 [7]. Furthermore, the WHO data showed that 52 million children under age five were suffering from wasting, and 155 million children were stunted in 2016 [7], representing a 10 million decrease from the data in 2011 [8]. In developing nations, the problem of nutrient deficiencies that manifest in undernutrition (underweight, wasting, and stunting) still persist, while the problems of overnutrition, such as overweight and obesity, have increased rapidly. The 2006 Food and Agriculture Organization (FAO) report referred to this phenomenon as a double burden of malnutrition, where under- and overnutrition occur simultaneously among different population subgroups in developing countries [9]. Based on the 2013 National Health Survey, the prevalence of the double burden of malnutrition is around 11% [4] in Indonesia, with some estimates much higher [10].

The double burden of malnutrition has raised public health concerns due to its consequences. The double burden will manifest through the deficiency diseases of undernutrition, while simultaneously leading to increases in the non-communicable diseases (NCDs) of overnutrition, such as obesity, cardiovascular disease, type 2 diabetes mellitus, and hypertension. The present amount of NCDs accounts for 80% of the total burden of disease mortality in developing countries; an estimated US $84 billion of economic production will be lost from heart disease, stroke, and diabetes alone [11]. Hence, the presence of the double burden of malnutrition will continue to burden the already inadequate and overextended health budget in developing countries [12].

Within the peer-reviewed literature, the term “double burden of malnutrition” varies in use among authors. Authors have addressed the double burden of malnutrition at the individual level [13], household level [14,15,16], and population or country level [17,18,19,20]. The double burden occurring within a household, as indicated by high prevalence of child stunting and overweight/obese mothers (SCOWT), has been deemed largely preventable, due to mothers and children sharing the same socioeconomic environment. Moreover, maternal parenting has been portrayed as a key factor to prevent SCOWT, as mothers often control the purchase and distribution of food in the household [21]. A study in Indonesia revealed that in households experiencing the double burden of malnutrition, women were less empowered and less involved in the decision-making process, resulting in higher nutrition and health inequality when compared to normal households [22]. Hence, we hypothesize that empowering women and involving them in the decision-making process for household matters, including food purchasing, could potentially prevent double burden of malnutrition. In this scenario, the role of the mother may be pivotal, but is also based on an assumption that households are not food insecure, limiting access to adequate food, let alone foods enabling a healthy diet.

The evidence shows that food insecurity is one of the risk factors for child stunting, but there is currently little evidence that food insecurity is risk factor for the double burden of malnutrition [5,15]. A cross-sectional study in rural Indonesia demonstrated that higher intakes of animal products was protective against SCOWT [10]. A Guatemalan study revealed that households suffering from SCOWT had the highest per capita animal protein consumption in the third quintile [15] and not in the lowest quintile. The first quintile of per capita consumption indicates that the consumption among households in this group was lowest compared to other households. One might expect that since their per capita consumption was the worst, problems that include stunting only or SCOWT would likely be the highest in this group, as an indication of limited food access. The problem of SCOWT, however, was the highest in the middle (third) quintile group, which has higher per capita consumption than the first quintile group. This was the opposite case to households with child stunting alone, where the per capita food consumption was in the first quintile [15]. Hence, these SCOWT households are believed to have some degree of food security that enables them to be placed in the middle quintile of per capita food consumption. Such evidence has led researchers to hypothesize that food insecurity is not associated with the double burden of malnutrition [15], as it was strongly associated with child stunting [5,15]. In this study, we aimed to determine the relationship between household food insecurity, as measured by the categorization of the household food insecurity access scale (HFIAS) and the prevalence of the double burden of malnutrition, as indicated by household’s with a stunted child and an overweight/obese mother (SCOWT).

## 2. Materials and Methods

**Figure A1:**
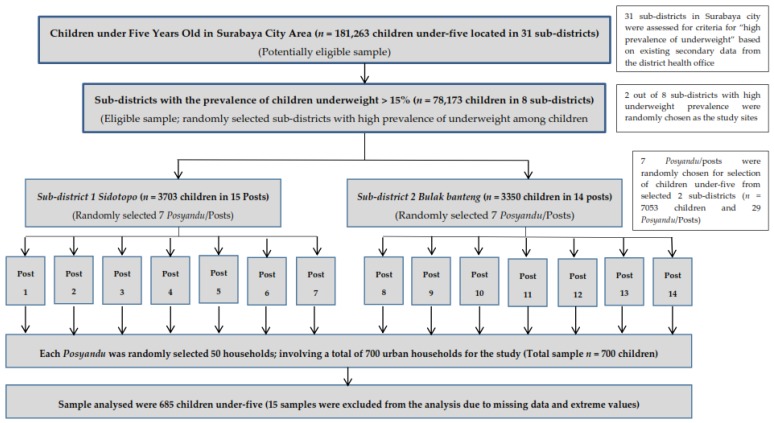
Systematic cluster sampling methods from 14 different integrated health posts (*posyandu*) involving total of 700 urban households in Surabaya, Indonesia.

This cross-sectional study of food security and the double burden of malnutrition was undertaken with individual assessments administered by trained interviewers in April to May 2015. Urban households were selected using systematic cluster sampling methods from 14 integrated health posts (“*posyandu*”) in Surabaya, Indonesia (Figure A1). Access to the existing secondary data of a monthly child growth monitoring system was given upon approval from the District Health Department in Surabaya, Indonesia. Based on these data, we determined a list of sub-districts in Surabaya City that had high prevalence of child underweight relative to the national prevalence. Sub-districts with a prevalence of child underweight of more than 15% were randomly selected as survey locations. In each sub-district, randomization was performed to select the community health post, so-called “*posyandu*” [23], as the point of anthropometric measurement. Mothers who came for a monthly child health monitoring at the “*posyandu*” were asked to participate in the study and provide informed consent. The inclusion criteria were: informed consent obtained, mother reported no physical disability to walk for minimum 10 min continuously, and mother had a child under five years old.

The power for the study was 82.5% designated to the prevalence of SCOWT, with a 95% confidence interval or 5% alpha level and the population estimate of children under five years old in Surabaya City used 2013 data (*n* = 181,263 children) [24]. Assuming a 50% response distribution, the minimal sample needed was 662 participants. Accounting for a 5% non-response rate, we surveyed 700 households with mothers and at least one child between two and five years of age. Excluding cases with missing data or extreme values [25], 685 households were analyzed.

A survey questionnaire was administered by a trained research assistant for a one-on-one interview in the mother’s house. The interview lasted approximately 30 min. The questionnaire consisted of demographic characteristics; socioeconomic status (based on the Indonesian Basic Health Research Questionnaire/IBHRQ); and food security, using the Household Food Insecurity Access Scale (HFIAS). All questionnaires were translated into the Indonesian language and the survey was delivered in a one-on-one interview using the Indonesian language (Bahasa). The HFIAS score ranged from absolute food security (score = 0) to severely insecure (maximum score = 27). There were four categories of food insecurity status, according to the HFIAS guidelines [26]: “food secure”, “mildly food insecure”, “moderately food insecure” and “severely food insecure”. A reliability analysis was performed to test the internal consistency for the nine HFIAS questions.

Anthropometric measurement was conducted in the “*posyandu*” by a trained research assistant, including maternal weight, maternal height, child weight, and child height/length. Child height and weight were assessed (in light clothing) using a stadiometer SECA 213 (Seca GmbH & Co. Kg, Hamburg, Germany) and a Camry EB6571 digital scale (Camry Electronic Ltd., Guangdong, China) to 0.01 kg for weight. Maternal weight and height were assessed (in light clothing) using a Camry EB6571 digital scale and height rod (stadiometer SECA 213, Seca GmbH & Co. Kg, Hamburg, Germany). Mothers were weighed and measured for height to determine obesity status. Child age in months was assessed using two sources, first from the mother’s answers when interviewed, and second based on the date of birth listed on the health monitoring card/registry in “*posyandu*”. If the month did not match, we used the “*posyandu*” registry as the primary source. Child stunting was defined as a *z*-score less than −2 standard deviations (SD) from the average height for age *z*-score (HAZ) based on the Multiple Growth Reference Standard (MGRS) of the WHO in 2006 [27]. Quality management of the HAZ data was applied using the cut-off for extreme values recommended by the WHO [25]. Children with a HAZ of more than ±6.0 were excluded from the analysis. Maternal BMI was calculated based on the BMI formula using the appropriate cut-off for an Asian population, with overweight defined as BMI of 23.0 kg/m^2^ to 27.4 kg/m^2^ and obese as a BMI of ≥27.5 kg/m^2^ or more. Finally, the double burden of malnutrition, as measured by SCOWT, was defined by the combined occurrence of child stunting and maternal overweight/obesity within one household.

We used a conventional Cronbach’s alpha of 0.65 to indicate that the questions in the HFIAS had an acceptable internal consistency [28]. Descriptive statistics were used to illustrate household characteristics and to determine the prevalence of the outcome variables. Potential predictors of child stunting, maternal overweight/obesity and SCOWT were determined by univariate logistic regression, including maternal literacy, maternal education, family type, number of children and number of children under the age of five years in the household, maternal occupation, paternal occupation, household monthly income, household food expenditure, paternal smoking status, and food insecurity status. Prior to the analysis, multicollinearity tests were performed for all independent variables listed above. We employed a variance inflation factor (VIF) of less than 2.5 for all our analyses to determine that multicollinearity was not a problem [29,30,31]. A Chi-squared test was performed to analyze the differences between household food insecurity status with the number of children under five, paternal occupation, monthly income, and paternal smoking status. Multiple logistic regression was performed to control for confounding factors (the number of children under five, paternal occupation, monthly income, and paternal smoking status) using backward stepwise selection with significance level of 0.2 for removal from the model. The backward stepwise selection was reliable in identifying useful predictors during the exploratory stages of model building. The difference in monthly income between various household food security statuses was tested using analysis of variance (ANOVA).

All data analysis was performed in IBM SPSS Statistics 22 (IBM, Armonk, NY, USA).

This study was approved by the Institutional Review Board (IRB) of Kansas State University, USA (reference or proposal number: 7646). In addition, this study was approved by the Surabaya City Review Board (Bakesbangpol No.: 1366/LIT/2015) in Indonesia. We explained the study objectives and obtained written informed consent during monthly integrated health post meetings (“*posyandu*”), where mothers bring their children under the age of five years for growth monitoring. Participants were free to withdraw from the study at any time without any consequences.

## 3. Results

The selected characteristics of the samples in this study are presented in Table A1. When we employed the Chi-squared test based on the food insecurity status of the households, significant differences in the household characteristics emerged for maternal literacy (*p* = 0.012) and maternal education (*p* < 0.0001), but not maternal occupation (*p* = 0.290). Most of the households were nuclear families, with 1–2 children and only one child under five years living in the household. Almost all fathers were working and nearly 70% of them were smokers. We obtained a Cronbach’s alpha of 0.831, which indicated that the nine questions of the HFIAS for the 685 households had sufficient internal consistency.

Chi-squared tests also showed significant differences in the household’s food insecurity status for the number of children under five in the household (*p* = 0.046), paternal occupation (*p* < 0.0001), monthly income (*p* < 0.0001), and paternal smoking status (*p* = 0.045). The ANOVA test showed that compared to the food secure households, households with some level of food insecurity were significantly different in terms of monthly income.

No significant difference, however, was found among households across food insecurity status in father’s monthly cigarettes expenses as well as food expenditure. Possession of electricity did not differ between households with different food insecurity statuses.

There were significant differences, however, among households with different food insecurity statuses in the possession of a radio/tape recorder (*p* < 0.0001), television (TV) (*p* = 0.013), telephone/hand phone (*p* < 0.0001), and fridge (*p* < 0.0001). The mean HFIAS score was 4.85 (SD: 5.6), with a range from 0 to 24. Table A2 shows the affirmative responses for the HFIAS items. More than half of the participants were worried about food, and nearly half of households had concerns that they were unable to eat preferred foods (47.4%), ate few kinds of foods (36.4%), and ate foods they really did not want to eat (35.5%).

Approximately 3.2% of the participants stated that in the last four weeks they, or any household member, went a whole day and night without eating anything. The majority of households never experienced a complete lack of food of any kind in the household for the past month (88.5%). Based on the HFIAS guidelines, many households were categorized as food secure (42%), but there were relatively high proportions of mild (22.9%), moderate (15.3%) and severe (19.7%) food insecurity.

The results revealed that when using the cut-off point of a BMI ≥ 25 kg/m^2^ as overweight, the prevalence of a double burden in the mother–child pair was 21.2%, whereas the prevalence of maternal overweight/obesity was 58.8%. When using the BMI cut-off point for an Asian population with BMI ≥ 23 kg/m^2^ as defining maternal overweight, the prevalence rose to 70.3%, and the double burden of malnutrition measured as SCOWT was 24.7%, while the prevalence of child stunting was 36.5%. The following results were set to use the cut-off point for overweight recommended for an Asian population.

As seen in Table A3, several variables were significantly associated with a double burden of malnutrition measured as SCOWT when tested using unadjusted logistic regression. They were: maternal height (OR = 0.893; 95% CI = 0.848–0.940), maternal education (OR = 0.565; 95% CI = 0.340–0.937), number of children (OR = 1.852; 95% CI = 1.184–2.898), paternal occupation, and food security. Compared to having their father working with a steady income as a government officer (including army and police), having a father who worked in a job with less steady income, such as in the private sector (OR = 3.963; 95% CI = 1.050–14.961), or in a trade or as an entrepreneur (OR = 4.840; 95% CI = 1.230–19.050), as labor (OR = 5.770; 95% CI = 1.542–21.586), or others (OR = 7.436; 95% CI = 1.871–29.549) increased the risk of SCOWT in the household. Likewise, similar patterns were observed in relation to SCOWT where the risk of SCOWT was increased in mildly food insecure (OR = 2.647; 95% CI = 1.486–4.712), moderately food insecure (OR = 2.254; 95% CI = 1.170–4.342) and severely food insecure households (OR = 2.057; 95% CI = 1.112–3.804) relative to food secure households. Based on the unadjusted model, the strongest predictors of SCOWT was paternal occupation, followed by food insecurity status and number of children. However, after adjustment for potential confounding variables such as maternal height and SES, the stepwise multiple logistic regression model for SCOWT revealed that only food insecurity status served as a significant predictor. Compared to food-secure households, mild food insecurity (aOR = 2.798; 95% CI = 1.540–5.083), moderate food insecurity (aOR = 2.530; 95% CI = 1.286–4.980), and severe food insecurity (aOR = 2.045; 95% CI = 1.087–3.848) increased the likelihood of households experiencing SCOWT.

The results of the univariate logistic regression showed that some variables were protective against stunting, namely maternal height (OR = 0.884; 95% CI = 0.842–0.927), child’s gender, maternal education (OR = 0.534; 95% CI = 0.342–0.834), paternal occupation, and food insecurity status. Female children were less likely to be stunted than their male counterparts (OR = 0.612; 95% CI = 0.441–0.849). Maternal education also showed a significant association with stunting; using mothers with low education level as a reference, having an educated mother lessened the likelihood of child stunting (OR = 0.534; 95% CI = 0.342–0.834). Similarly, educated mothers showed a protective effect on SCOWT (OR = 0.565; 95% CI = 0.340–0.937) but not highly educated mothers. For paternal occupation, the risk of child stunting was increased in the households where the father was working in the private sector (OR = 4.914; 95% CI = 1.476–16.360), in trade or as an entrepreneur (OR = 7.274; 95% CI = 2.099–25.208), as labor (OR = 7.196; 95% CI = 2.140–24.201), or others (OR = 11.117; 95% CI = 3.157–39.153), compared to fathers who worked as government officers (including army, police). Using food secure households as a reference, increased likelihood of child stunting was observed in households with mild food insecurity (OR = 1.740; 95% CI = 1.043–2.903), and severe food insecurity (OR = 2.182; 95% CI = 1.280–3.717). The stepwise multiple logistic regression models showed that child gender, maternal education and food insecurity status were significantly associated with child stunting. Factors that were significantly associated with a reduced likelihood of child stunting were child gender (aOR = 1.696; 95% CI = 1.077–2.672), and educated mother (aOR = 0.596; 95% CI = 0.372–0.954). Compared to the food secure households, only households that were severely food insecure increased the likelihood of child stunting (aOR = 2.005; 95% CI = 1.140–3.526). Having 3–4 children, relative to having 1–2 children at home, was associated with an increased likelihood of maternal overweight/obesity (aOR = 1.750; 95% CI = 1.108–2.762).

## 4. Discussion

The objective of our study was to analyze the relationship between household food insecurity, as measured by the household food insecurity access scale (HFIAS) category, and the double burden of malnutrition, as indicated by household’s with a stunted child and an overweight/obese mother (SCOWT). These results support the hypothesis that the double burden of malnutrition is robustly related to food insecurity, and the categorization of the HFIAS as a measure of food insecurity is a predictor of SCOWT. To the best of our knowledge, this is the first published study on the application of this scale within an urban setting in Indonesia.

Our study, based in an urban setting, revealed that the prevalence of the double burden in mother–child pairs was 24.7%, whereas the prevalence of child stunting was 36.4%, and maternal overweight/obesity was 70.2%. This prevalence was higher than that found in a previous study in a rural Indonesian setting that found 11% of the sample consisted of maternal overweight and a stunted child coexisting within the same household [16]. A more recent study in a rural setting of Indonesia reported a higher percentage of double burden, as measured by the coexistence of maternal overweight and child stunting in one household [10]. A staggering 30.6% of the double burden in mother–child pairs was reported in that study [10], where maternal overweight was set at a BMI above 23.5 kg/m^2^, while our study used the conventional threshold of above 25.0 kg/m^2^ In an Indonesian setting, regional differences were argued to explain the discrepancy in the prevalence of double burden observed in mother–child pairs in urban and rural areas [10,22]. Studies in Nairobi, Kenya showed that the obesogenic environment in an urban setting was characterized by reliance on energy-dense street food and was arguably responsible for the rise of overweight/obesity in this population [32,33]. Unfortunately, we did not collect data on energy-dense street food to be able to make comparisons of our findings with studies from Nairobi, Kenya. Nevertheless, evidence from studies in Indonesia has consistently shown a prevalent problem of the double burden of malnutrition in the form of stunted child and overweight/obese mother pairs across settings and geographical locations. Comparing with previous studies in rural settings, our results from an urban setting have a higher prevalence of the double burden of malnutrition, hence supporting the hypothesis that the double burden of malnutrition is more prevalent in urban areas. These findings have the policy implication that people in urban areas should be educated on the concept of energy balance, improving healthy eating and physical activity through health and nutrition education, as well as environmental conditioning that supports health. On the other hand, the rate of urbanization should be decreased by means of improving the living conditions and employment in rural areas.

Although evidence showing a relationship between food insecurity and child stunting is abundant, there is a scarcity of evidence relating to food insecurity with a double burden of malnutrition. In this study, we found that food insecurity was significantly associated with the double burden of malnutrition, as observed in the coexistence of stunted children and overweight/obese mothers within the same household. Most of the households involved in this study were experiencing some form of food insecurity (58%) as defined by the HFIAS. This is not surprising, as 11% of Indonesia’s 252 million people live below the national poverty line of $1 income per day, with an additional 40% hovering marginally above the line. Studies have shown that poverty is closely linked with food insecurity. In a univariate logistic regression, using food secure households as a reference, we revealed that having a mildly food insecure household increased the risk of a double burden by more than three times, having a moderately food insecure household increased the risk by more than three times, and having a severely food insecure household increased the risk by more than two times. This association persisted in the multivariate logistic model; in fact, the likelihood was even stronger. Compared to the food secure households, a mildly food insecure household showed a risk of double burden more than 3.7 times higher. Having a moderately food insecure household increased the risk by 4.5 times, and having a severely food insecure household increased the risk by more than three times.

Our study showed that food insecurity was more robust in predicting the double burden of malnutrition indicated by SCOWT than in predicting child stunting alone. As seen in Table A3, even though four categories of food insecurity were significantly correlated to both SCOWT and child stunting in the univariate logistic regression, only severely food insecure households remained associated with child stunting in the multivariate model, while any form of food insecurity remained significantly associated with SCOWT in the multivariate logistic regression model. We are not aware of any published studies with which to compare our findings that relate food insecurity and the double burden of malnutrition. A study in Indonesia highlighted an association between living in households with a higher socioeconomic status (SES) and an increased risk of the double burden of malnutrition [22]. A study in Guatemala also showed an association between per capita household consumption and SCOWT [15]. Both of these studies revealed that the double burden of malnutrition was related to some form of household access to food, indicated by higher SES and per capita consumption. The fact that our analysis, using levels of food insecurity status, showed a steady association with SCOWT and not with child stunting aligns with this argument. Mildly food insecure and moderately food insecure households were significantly associated with an increased risk of SCOWT, and not significantly correlated with child stunting. This was an indication that lower quality and choice of food as a building block for mildly and moderately food insecure households were sensitive enough to predict SCOWT.

In broader terms, we believe that with minor food insecurity, households may compromise their diet towards cheaper food that is mostly high in energy. Hence, we argue that the double burden of malnutrition exists because the children were stunted due to insufficient availability and intake of growth-promoting and nutrient-dense foods; while mothers were supplied with an abundance of energy-dense foods that promote weight gain. In countries experiencing a nutrition transition, much of the energy-dense food is not nutritionally dense, and hence provides limited support for children’s growth [14]. Households that were not facing mild and moderate food insecurity might be able to purchase foods that are more expensive but more nutrient-dense, such as animal-based foods. Animal-based foods are a good source of growth-promoting nutrients, such as protein [34]. A study in rural Indonesia showed that a dietary pattern of “high-animal products” was associated with a decreased likelihood of SCOWT, through a strong inverse correlation with child stunting [10].

The present study’s findings also add evidence regarding the relationship between maternal height/stature and the double burden of malnutrition. A similar significant association, however, was seen for maternal height and child stunting alone. Hence, the observed correlation between maternal height and SCOWT might be driven by the association between maternal height and child stunting, even though the adjusted odds was slightly stronger for SCOWT. It is established that maternal height is a genetic predictor for child’s height that later defines stunting. For this reason, we believe that compared to food insecurity status as measured by the HFIAS category, maternal height was less robust in predicting SCOWT. Regardless, our study extended the widely-reported association between maternal short stature and child stunting in developing countries such as Bangladesh [16], Brazil [35,36], Indonesia [16] and Mexico [37].

The multivariate logistic regression revealed that households with 3–4 children were more likely to have mothers who were overweight/obese, compared to households with 1–2 children. With less than one-third of mothers exclusively breastfeeding in Indonesia [9,38], it is probable that repeated pregnancy increases the risk for maternal overweight/obesity. Mothers who provide their children with exclusive breastfeeding for three months had four times greater weight loss, compared to mothers who did not exclusively breastfed or who discontinued breastfeeding their child before three months [39]. Household spending of US $100–150 on food per month also increased the risk of maternal overweight/obesity, compared to households with monthly food expenditures of less than US $50. Spending US $50–100 as well as >US $150 was not significantly related to maternal overweight/obesity. This indicates that spending > US $100–150 was the range of monthly food expenses that may contribute to a maternal energy imbalance through the purchase of energy-dense food. However, both variables (number of children and food expenditure) were not significantly correlated with SCOWT.

The strengths of our study were the relatively large sample derived from a fully-powered sample size calculation, random sample, validated food insecurity instrument, and one-on-one interview by trained interviewers to complete questionnaires with the mother. In addition, compared to a recent study on the mother–child double burden in rural Indonesia, this study employed a conventional cut-off point for maternal overweight that was easily comparable to the body of knowledge related to the household-level double burden of malnutrition.

A few limitations should be noted for this study. First, since it was a cross-sectional study, a causal inference for food insecurity status and the double burden of malnutrition as observed in SCOWT cannot be established. An observational cohort study that examines changes in food insecurity over time would add more weight to the evidence for a positive relationship between food insecurity status as measured in the HFIAS category and SCOWT. Second, although the HFIAS was designed for cross-cultural settings, it was possible that some of the local perspectives on food insecurity failed to be captured in the questions. We minimized errors by excluding extreme values according to the recommended cut-off for the height-for-age *z*-score of the WHO [25]. Our results may be limited by reliance on multiple logistic regression with adjustment for the clustering of the samples using a mixed effect model; it is possible that a generalized estimating equation analysis (GEE) might produce more robust results, but it is unlikely that the direction and size of observed relationships would change in meaningful ways. Last, our first attempt to choose the study site by restricting it to only sub-districts with a prevalence of child underweight of more than 15% according to government reports might limit generalization to the general population.

## 5. Conclusions

In conclusion, one fifth of households in the study site were found to be experiencing the double burden of malnutrition in the form of a stunted child and an overweight/obese mother (SCOWT). The present study supported the hypothesis that the double burden of malnutrition is related to food insecurity. Even though both maternal height and household food insecurity status was significantly associated with the double burden of malnutrition, only the level of food insecurity derived from the HFIAS instrument served as a good predictor of SCOWT in urban Indonesia. Future studies could build on our results using the universally acceptable HFIAS instrument as an early warning indicator for the double burden of malnutrition as defined by the coexistence of maternal overweight/obesity and child stunting. These results should be replicated for different populations and settings to established HFIAS as the common predictor of the double burden of malnutrition and not merely as a food security indicator.

## Appendix B

**Table A1:** Characteristics of households with children between 2 and 5 years old from communities with a high risk of underweight children in Surabaya, Indonesia on their food security status.

Variable	Food Security		Mild Insecurity		Moderate Insecurity		Severe Insecurity		Total		*p*
	*n*	Row %	*n*	Row %	*n*	Row %	*n*	Row %	*n*	Column %	
Total	288	42.0	157	22.9	105	15.3	135	19.7	685	100	
Maternal literacy											0.012 *
Illiterate	17	29.8	11	19.3	9	15.8	20	35.1	57	8.3	
Partially literate	32	34.0	20	21.3	19	20.2	23	24.5	94	13.7	
Literate	239	44.8	126	23.6	77	14.4	92	17.2	534	78.0	
Maternal education											<0.001 ***
Low (no schooling or elementary school)	88	27.5	83	25.9	54	16.9	95	29.7	320	46.8	
Medium (junior high school)	62	44.9	32	23.2	27	19.6	17	12.3	138	20.1	
High (senior high school or college/university)	138	460.8	42	18.5	24	10.6	23	10.1	227	33.1	
Number of children under 5 years old in the household											0.046 *
1 child	260	42.1	141	22.9	91	14.8	125	20.3	617	90.1	
2 children	28	43.1	13	20	14	21.5	10	15.4	65	9.5	
Maternal occupation											0.290
Housewife without maid	216	41.5	118	22.7	86	16.5	100	19.2	520	75.9	
Housewife with maid	10	38.5	5	19.2	4	15.4	7	26.9	26	3.8	
Private sector	23	54.8	7	16.7	4	9.5	8	19.1	42	6.1	
Trade and entrepreneur	25	49.0	11	21.6	5	9.8	10	19.6	51	7.5	
Labor/miscellaneous services	11	25.6	16	37.2	6	14.0	10	23.3	43	6.3	
Paternal occupation (*n* = 650)											<0.001 ***
Government officer (including Army/Police)	56	88.9	4	6.4	2	3.2	1	1.6	63	9.7	
Private sector	93	48.4	49	25.5	24	12.5	26	13.5	192	29.5	
Trade and entrepreneur	44	39.6	26	23.4	28	25.2	13	11.7	111	17.1	
Labor	62	31.5	53	26.9	25	12.7	57	28.9	197	30.3	
Other	23	26.7	18	20.9	22	25.6	23	26.7	86	13.2	
Household’s monthly income (*n* = 508)											<0.001 ***
Low (≤Indonesian Rupiah (IDR) 1,500,000 or≤$150)	74	28.3	71	27.1	48	18.3	69	26.3	262	51.6	
Medium (>IDR 1500,000–2,500,000 or >$150–250)	52	42.6	22	18.0	20	16.4	28	23.0	122	24.0	
High (>IDR 2,500,000 or >$250)	93	75.0	19	15.3	4	3.2	8	6.5	124	22.4	
Paternal smoking status (*n* = 683)											0.045 *
Non-smoker	102	50	42	20.6	25	12.3	35	17.2	204	29.8	
Smoker	184	38.5	114	23.9	80	16.7	100	20.9	478	69.8	

Variables are significantly associated with food security status: * *p* < 0.05, ** *p* < 0.01, *** *p* < 0.001, *p*-values obtained with chi-squared tests and Fisher’s exact test for small cell sizes (less than 10 participants), *n* = 685 unless indicated otherwise.

**Table A2:** Distribution of affirmative responses and mean scores to items on the Household Food Insecurity Access Scale (HFIAS): households (*n* = 685) in urban Surabaya, Indonesia, May 2015.

HFIAS Questions (Due to Lack of Food or Limited Resources to Obtain Food, in the Past Four Weeks Did You or Any Household Member…)	Affirmative Responses	
	*n*	%
Q1: Worry about food	353	51.5
Q2: Unable to eat preferred foods	325	47.4
Q3: Eat just a few kinds of foods	249	36.4
Q4: Eat foods they really do not want to eat	243	35.5
Q5: Eat small meals a day	199	29.1
Q6: Eat fewer meals in a day	156	22.8
Q7: No food of any kind in the household	79	11.5
Q8: Go to sleep hungry	104	15.2
Q9: Go a whole day and night without eating	22	3.2

HFIAS, Household Food Insecurity Access Scale. Q, question.

**Table A3:** Odds ratios (ORs) for child stunting, maternal overweight/obesity, and SCOWT using simple and multiple logistic mixed models.

Variable	Child Stunting				Maternal Overweight/Obesity				SCOWT			
	Crude OR	95% CI	Adjusted OR	Adjusted 95% CI	Crude OR	95% CI	Adjusted OR	Adjusted 95% CI	Crude OR	95% CI	Adjusted OR	Adjusted 95% CI
Child Gender												
Male	Ref.		Ref.		Ref.				Ref.			
Female	0.612 **	(0.441–0.849)	1.696 **	(1.077–2.672)	1.16	(0.834–1.614)			0.74	(0.515–1.064)		
Child Age	0.989	(0.969–1.009)			1.014	(0.993–1.036)			0	-		
Maternal Age	0.996	(0.963–1.031)			1.026	(0.99–1.063)			1.02	(0.982–1.06)		
Maternal literacy												
Illiterate	Ref.				Ref.				Ref.			
Partially literate	0.994	(0.47–2.105)			0.897	(0.395–2.037)			1.021	(0.457–2.28)		
Literate	0.702	(0.374–1.316)			0.826	(0.416–1.637)			0.782	(0.396–1.547)		
Maternal education												
Low education	Ref.		Ref.		Ref.				Ref.			
Educated	0.534 **	(0.342–0.834)	0.596 *	(0.372–0.954)	0.814	(0.537–1.235)			0.565 *	(0.34–0.937)		
Highly educated	0.58	(0.213–1.579)	0.735	(0.242–2.23)	0.717	(0.329–1.565)			0.849	(0.294–2.454)		
Family type												
Nuclear family	Ref.				Ref.				Ref.			
Extended family	0.976	(0.663–1.435)			0.901	(0.613–1.324)			1.291	(0.584–1.361)		
Number of child at home												
1–2 children	Ref.				Ref.		Ref.		Ref.			
3–4 children	1.48	(0.978–2.238)			1.75 *	(1.108–2.762)	1.75 *	(1.108–2.762)	1.852 **	(1.184–2.898)		
>4 children	1.381	(0.422–4.525)			0.473	(0.15–1.484)	0.473	(0.15–1.484)	1.229	(0.317–4.766)		
Number of children under 5 years in household												
1 child	Ref.				Ref.				Ref.			
2 children	0.987	(0.524–1.86)			0.745	(0.401–1.384)			1.015	(0.504–2.044)		
3 children	0.877	(0.054–14.25)			0.855	(0.052–13.963)			1.497	(0.091–24.713)		
Maternal occupation												
Housewife without maid	Ref.				Ref.				Ref.			
Housewife with maid	1.472	(0.508–4.267)			2.374	(0.572–9.859)			1.935	(0.653–5.733)		
Private sector	0.435	(0.158–1.198)			1.41	(0.536–3.705)			0.795	(0.286–2.212)		
Trade and entrepreneur	0.772	(0.339–1.756)			0.734	(0.334–1.611)			0.686	(0.262–1.798)		
Labor/miscellaneous	0.719	(0.286–1.807)			1.245	(0.485–3.193)			0.817	(0.294–2.274)		
Paternal occupation												
Government officer	Ref.				Ref.				Ref.			
Private sector	4.914 **	(1.476–16.36)			0.97	(0.441–2.135)			3.963 *	(1.05–14.961)		
Trade and entrepreneur	7.274 ***	(2.099–25.208)			0.904	(0.386–2.12)			4.84 *	(1.23–19.05)		
Labor	7.196***	(2.14–24.201)			1.455	(0.654–3.239)			5.77 **	(1.542–21.586)		
Other	11.117 ***	(3.157–39.153)			0.977	(0.395–2.414)			7.436 **	(1.871–29.549)		
Paternal smoking status												
Non–smoker	Ref.				Ref.				Ref.			
Smoker	0.97	(0.677–1.389)			1.081	(0.756–1.546)			0.973	(0.652–1.451)		
Food insecurity												
Food secure	Ref.		Ref.		Ref.				Ref.		Ref.	
Mildly food insecure	1.74 *	(1.043–2.903)	1.687	(0.985–2.889)	1.286	(0.76–2.176)			2.647 ***	(1.486–4.712)	2.798 ***	(1.54–5.083)
Moderately food insecure	1.514	(0.84–2.729)	1.562	(0.842–2.897)	1.174	(0.646–2.135)			2.254 *	(1.17–4.342)	2.53 **	(1.286–4.98)
Severely food insecure	2.182 **	(1.28–3.717)	2.005 *	(1.14–3.526)	1.111	(0.647–1.91)			2.057 *	(1.112–3.804)	2.045 *	(1.087–3.848)

Households with children between 2 and 5 years old from communities with high risk of underweight children in Surabaya, Indonesia. Odds from those food-secure households. * *p* < 0.05, ** *p* < 0.01, *** *p* < 0.001; *p*-values and estimated values obtained by fitting a Mixed Effect Model accounting for Posyandu; Adjustment for Multiple Comparisons: Dunnett-Hsu.
